# Exploring the mechanisms underlying quercetin, a key component of *Achyranthis Bidentatae* Radix, against intervertebral disc degeneration

**DOI:** 10.3389/fimmu.2026.1744969

**Published:** 2026-03-10

**Authors:** Xin Liu, Xinrui Song, Yongbin Wang, Jingwei Zhang, Songrui Zhang, Lihuang Cui, Weitao He, Zhibin Geng, Xintao Wang

**Affiliations:** 1 Department of Orthopedic Surgery, The Second Affiliated Hospital of Harbin Medical University, Harbin, China; 2 Department of Spinal Surgery, The Fifth Hospital of Harbin, Harbin, China; 3 Harbin Medical University, Harbin, China

**Keywords:** *Achyranthis bidentatae* Radix, intervertebral disc degeneration, network pharmacology, PI3K/Akt/eNOS pathway, quercetin

## Abstract

**Objective:**

Intervertebral disc degeneration (IDD) is a complex, multifactorial orthopedic disorder. This study aims to investigate the therapeutic effects and underlying mechanisms of quercetin (QUE), a key bioactive component of *Achyranthis Bidentatae* Radix (ABR), against IDD.

**Methods and results:**

Network pharmacology and RNA sequencing (RNA-seq) were utilized to identify active components and key molecular targets of ABR in IDD treatment. The findings indicated that 30 overlapping hub genes were enriched in pathways associated with hypoxia, collagen biosynthesis, inflammation, apoptosis, angiogenesis, and PI3K-Akt signaling. Network construction, molecular docking, and molecular dynamics (MD) simulation revealed that QUE, a major bioactive component of ABR, exhibits strong binding affinity to *NOS3* (eNOS). An *in vitro* IDD model was established using nucleus pulposus (NP) cells stimulated with interleukin-1β (IL-1β). QUE significantly improved NP cell viability and mitigated IL-1β-induced oxidative stress, extracellular matrix (ECM) degradation, inflammation, apoptosis, and cellular senescence. Additionally, QUE suppressed PI3K, Akt, and eNOS phosphorylation, suggesting its role in modulating IDD progression. Mechanistically, loss-of-function validation confirmed *Nos3* as an essential component within this pathway. Functional assessment further demonstrated that QUE significantly reduced IL-1β-induced NO overproduction in NP cells, confirming its regulatory effect on the PI3K/Akt/eNOS pathway. Finally, *in vivo*, QUE attenuated the degree of IDD in the puncture-induced rat model.

**Conclusions:**

Our results demonstrate that QUE, a key active component of ABR, exerts protective effects on NP cells by alleviating IL-1β-induced oxidative stress, ECM degradation, inflammation, apoptosis, and senescence. These effects may be mediated through the PI3K/Akt/eNOS signaling pathway, with *Nos3* serving as an indispensable downstream component. Our findings elucidate a novel mechanism of QUE and provide a pharmacological basis for the therapeutic application of ABR in IDD management.

## Graphical Abstract

**Figure:**
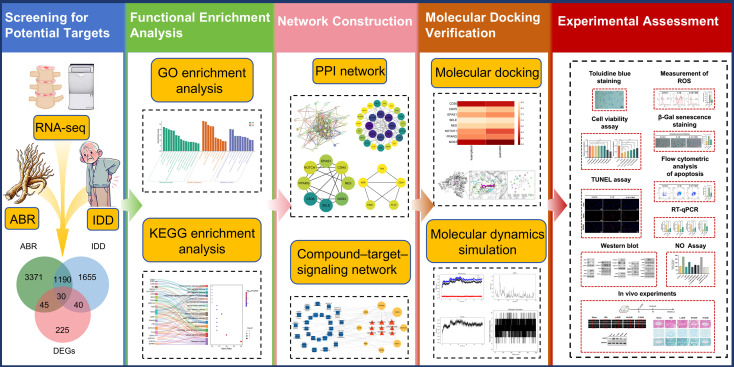

## 1 Introduction

Intervertebral disc degeneration (IDD) is a complex, multifactorial orthopedic disorder that leads to disability and imposes a significant economic burden worldwide (1). Disc inflammation, extracellular matrix (ECM) disruption, and ingrowth of blood vessels are the most important pathological changes in IDD (2). The intervertebral disc (IVD) consists of a gel-like nucleus pulposus (NP), collagen-rich annulus fibrosus (AF) layers, and cartilaginous endplates (CEP), all of which are crucial for spinal flexibility and biomechanical integrity (3). NP cells, located in the inner core of the IVD, play a key role in maintaining ECM homeostasis (4). Among proinflammatory cytokines implicated in IDD, interleukin-1β (IL-1β) is particularly significant, correlating with greater histological severity of degeneration. IL-1β promotes oxidative stress, inflammation, ECM degradation, apoptosis, and cellular senescence, thereby accelerating IDD progression (5, 6). Consequently, targeting IL-1β signaling represents a promising therapeutic approach for IDD.

In Traditional Chinese Medicine (TCM), IDD and associated low back pain (LBP) are classified under “Bi Syndrome” (7), which is attributed to pathogenic influences such as wind, cold, dampness, and heat. These factors obstruct meridians and impair qi and blood circulation, leading to pain and dysfunction (8). Strengthening the kidneys and lower back is fundamental to improving circulation and alleviating symptoms (9). Various herbal compound prescriptions, including Yi-Qi-Huo-Xue-Tang, Liuwei Dihuang decoction, Duhuo Jisheng decoction, and Bu-Shen-Huo-Xue-Fang, are traditionally used to treat IDD and LBP, with *Achyranthis Bidentatae* Radix (ABR) being a key herb in these formulations (10–12). According to the TCM classic “Ben Cao Gang Mu”, ABR has the therapeutic effects of promoting blood circulation, nourishing the liver and kidneys, and strengthening the tendons and bones (13). Based on the TCM theory of IDD, ABR demonstrates promising therapeutic potential for the treatment of IDD.

ABR (Chinese: Niu Xi; Scientific: *Achyranthes bidentata* Blume), a member of the Amaranthaceae family, is traditionally recognized for its ability to nourish the liver and kidneys, enhance circulation, and strengthen bones and tendons (14). It contains a diverse array of bioactive compounds, including polysaccharides, peptides, alkaloids, organic acids, saponins, ketosteroids, and trace elements (15). Modern chemical analyses further specify that quercetin (QUE) is one of the abundant and critical flavonoid active components in ABR, providing a concrete chemical basis for investigating its role (16, 17). Research has increasingly focused on its regulatory effects on bone metabolism, along with its anti-inflammatory and antioxidant properties (18, 19). ABR has demonstrated therapeutic potential in rheumatoid arthritis, osteoporosis, osteoarthritis, and bone injury, suggesting its broader clinical relevance (13, 14). While preliminary evidence indicates that ABR may be beneficial in treating IDD, further research is warranted to elucidate its precise mechanisms.

Network pharmacology, an emerging interdisciplinary approach, facilitates the investigation of drug actions and drug-disease interactions at a systems level (20). This method aligns well with the holistic principles of TCM and provides a suitable framework for studying IDD treatment mechanisms (21). Additionally, RNA sequencing (RNA-seq) enables the identification of differentially expressed genes (DEGs), offering valuable insights into transcriptional responses to treatment (3, 22). In this study, we employed network pharmacology and RNA-seq to identify potential active components of ABR against IDD. The results pinpointed QUE as the key candidate, prompting subsequent *in vitro* experiments to validate its therapeutic role and mechanism. QUE exhibits strong binding affinity to *NOS3* (eNOS), suggesting that its protective effects may be mediated through the PI3K/Akt/eNOS pathway. Furthermore, a puncture-induced rat model was utilized to evaluate the protective effect of QUE on IDD.

## 2 Materials and methods

### 2.1 NP tissue samples

Human NP tissue samples were collected from six patients. Three patients (one male, two females; mean age: 43.67 ± 2.52 years; Grade IV–V) underwent endoscopic lumbar discectomy for lumbar disc herniation. The control group included three patients (two males, one female; mean age: 29.33 ± 6.11 years; Grade I–II) who underwent surgery for traumatic lumbar fractures. All procedures were performed at the Second Affiliated Hospital of Harbin Medical University (Harbin, China) between May and July 2024. Disc degeneration severity was independently assessed by three observers using the Pfirrmann grading system based on preoperative T2-weighted MRI. Ethical approval was obtained from the Second Affiliated Hospital of Harbin Medical University (No. YJSKY2024-402). **Supplementary Table 1** provides detailed clinical characteristics of the patients.

### 2.2 Identification of IDD targets

To identify IDD-associated targets, we searched GeneCard (https://www.genecards.org/) (23), OMIM (https://www.omim.org/) (24), and PharmGKB (https://www.pharmgkb.org/) (25) using the keyword “intervertebral disc degeneration”. GeneCards results were filtered by a relevance score > 1, and duplicate entries were removed to establish a comprehensive IDD gene set.

### 2.3 Identification of ABR active components and targets

The TCMSP database (https://www.tcmsp-e.com/) was used to identify the active ingredients of ABR (26). Selection criteria for pharmacokinetic properties included oral bioavailability (OB) ≥ 30% and drug-likeness (DL) ≥ 0.18 (27).

To identify potential targets of ABR’s active compounds, we queried multiple databases using “Homo sapiens” as the filtering condition: PubChem (https://pubchem.ncbi.nlm.nih.gov/) (28), PharmMapper (http://lilab-ecust.cn/pharmmapper/) (29), SwissTargetPrediction (http://www.swisstargetprediction.ch) (30), and BATMAN-TCM (http://bionet.ncpsb.org.cn/batman-tcm/index.php/) (31). The retrieved targets were standardized to gene symbols using the UniProt database (https://www.uniprot.org/) (32). After removing duplicates, we obtained the final set of potential ABR targets.

DEGs, ABR targets, and IDD targets were compared, and a Venn diagram was generated using an online bioinformatics platform (http://www.bioinformatics.com.cn/). This analysis identified 30 overlapping genes, which were selected as core targets for ABR-based IDD therapy in subsequent research. The provenance, key filtering criteria, and preprocessing steps for all datasets used in this bioinformatics analysis are summarized in **Supplementary Table 2**.

### 2.4 Functional enrichment analysis

The DAVID database (https://davidbioinformatics.nih.gov/) was utilized for Gene Ontology (GO) and Kyoto Encyclopedia of Genes and Genomes (KEGG) pathway enrichment analysis, with a significance threshold of *P* < 0.05 (33, 34).

### 2.5 Network construction

To construct the protein-protein interaction (PPI) network, overlapping targets were analyzed in the STRING database (http://string-db.org), applying a minimum interaction score of 0.400 (35). The resulting network was imported into Cytoscape (version 3.10.2) for visualization and further analysis (36). MCODE (version 2.0.3) was employed for clustering, identifying core targets based on Node Score Threshold = 0.2, K-Core = 2, and Max Depth = 100 (37). Core targets were those within the highest-scoring subnetwork.

We then constructed the component-target-pathway network in Cytoscape, where nodes represented ABR’s active compounds, target genes, and key pathways, while edges denoted interactions. CytoNCA (version 2.1.6) was used to calculate degree values, refining network visualization based on these values (38).

### 2.6 Molecular docking and molecular dynamics simulation

Compound structures were retrieved from PubChem, and energy minimization was conducted using Chem3D (version 17.0). The optimized structures were saved in PDB format, with rotatable bonds defined and stored as PDBQT files for docking. Protein structures were obtained from the RCSB PDB database (https://www.rcsb.org/) (39). The native ligand was removed using PyMOL, and the docking site was defined by specifying central coordinates (X, Y, Z) and dimensions. AutoDockTools (version 1.5.6) prepared the ligand-receptor system for semi-flexible docking using AutoDock Vina. Docking results were visualized in PyMOL, and protein-ligand interactions were analyzed via the PLIP server (https://plip-tool.biotec.tu-dresden.de/plip-web/plip) (40).

In this study, MD simulations were conducted using GROMACS 2022.3 to analyze the interaction between *NOS3* and QUE. Following a previously described protocol (41), the simulation workflow began with energy minimization, followed by equilibration under both isothermal-isovolumic and isothermal-isobaric conditions for 100,000 steps each. Each phase used a coupling constant of 0.1 ps and lasted 100 ps. The system then underwent a free MD simulation for 100 ns, comprising 5,000,000 steps with a 2 fs time step.

### 2.7 Cell extraction and cell culture

For biological validation, ten 6-week-old Sprague-Dawley rats were euthanized via intraperitoneal administration of a lethal dose of pentobarbital (150 mg/kg) and then disinfected. Lumbar vertebrae were aseptically collected, placed in 15-mL centrifuge tubes, and rinsed three times with PBS. Under an operating microscope, NP tissue was carefully extracted from the IVDs, minced, and digested in 0.25% trypsin-EDTA (Gibco, New York, USA) within a sealing membrane at 37 °C. Digestion continued until the tissue edges became blurred and the solution exhibited slight viscosity. The resulting cell suspension was transferred to DMEM/F12 (1:1) medium (Biosharp, Hefei, China) supplemented with 10% fetal bovine serum (FBS; ExCell Bio, Shanghai, China) and 1% penicillin/streptomycin (PS; Biosharp, Hefei, China). The suspension was filtered through a 70 μm sieve into centrifuge tubes and centrifuged at 300 g for 5 min. The supernatant was discarded, and the pellet was washed with PBS, recentrifuged, resuspended in 6 mL of culture medium, and transferred to culture tubes. After 2 weeks of aggregation, the cells were subcultured, and second-generation NP cells were used for subsequent experiments. All NP cells were maintained at 37 °C in a humidified atmosphere with 5% CO_2_.

### 2.8 RNA-seq

Total RNA was extracted from NP samples using TRIzol (Invitrogen, Carlsbad, CA, USA) for transcriptome sequencing. The workflow included RNA extraction, library construction, raw data quality assessment, and sequencing. DEGs were identified based on a fold-change threshold of >2 or <0.5 using a parametric F-test in the R package edgeR (*P* < 0.05). Functional enrichment analysis was performed using DAVID.

### 2.9 Toluidine blue staining

NP cells were seeded in 24-well plates and washed twice with PBS (1 min each). Toluidine blue staining (Solarbio, Beijing, China) was performed by incubating cells with the stain for 5 min, followed by the addition of an equal volume of distilled water with gentle shaking. After 15 min, the plates were rinsed twice with distilled water (30 s each) and fully submerged for microscopic examination.

### 2.10 Cell viability assay

Cell viability was assessed using the CCK-8 assay (Meilunbio, Dalian, China). Cells were digested, plated at 3 × 10³ cells/well in 96-well plates, and treated with IL-1β (MedChemExpress, Shanghai, China), LY294002 (MedChemExpress, Shanghai, China), and varying concentrations of QUE (MCE, New Jersey, USA) for 48 h. After treatment, 10 μL of CCK-8 reagent was added to each well, mixed, and incubated at 37°C in the dark for 1–4 h. Absorbance at 450 nm was measured using a microplate reader.

### 2.11 β-Gal senescence staining

β-Galactosidase staining for senescence was performed using a β-Gal staining kit (Solarbio, Beijing, China). NP cells were washed with PBS, fixed with 1 mL of β-Gal fixative for 15 min, and rinsed three times with PBS. Staining solution (1 mL) was then added, and cells were incubated overnight at 37°C in a sealed, humidified chamber in the dark before microscopic observation. SA-β-gal–positive cells, defined by intense blue−green cytoplasmic staining, were counted in a blinded manner by two independent observers, and the percentage of senescent cells was calculated as (number of positive cells/total cells) × 100% using the mean of the two counts for statistical analysis.

### 2.12 Measurement of ROS

ROS levels were measured using a ROS assay kit (Beyotime, Shanghai, China). The DCFH-DA probe was diluted to 10 μM in serum-free medium. NP cells were harvested, resuspended in the DCFH-DA solution at a density of 1 × 10^6^–2 × 10^7^ cells/mL, and incubated at 37°C for 20 min with gentle mixing every 3–5 min. After incubation, cells were washed three times with serum-free medium to remove uninternalized DCFH-DA. ROS levels were then quantified via flow cytometry.

### 2.13 Flow cytometric analysis of apoptosis

Apoptosis was assessed using an Annexin V-FITC assay kit (Beyotime, Shanghai, China). NP cells from each group were collected, and 195 μL of cell suspension (5 × 10^5^–10 × 10^5^ cells) was incubated with 5 μL of Annexin V-FITC and 10 μL of propidium iodide for 10–20 min at room temperature in the dark. Apoptotic cells were subsequently analyzed by flow cytometry.

### 2.14 TUNEL assay

For TUNEL staining, NP cells were fixed in 4% paraformaldehyde (Biosharp, Hefei, China) and permeabilized with 0.1% Triton X-100 (BioFroxx, Einhausen, Germany). Apoptotic nuclei were labeled using a TUNEL apoptosis detection kit (Roche, Basel, Switzerland), followed by DAPI staining. Fluorescent images were captured and analyzed via fluorescence microscopy.

### 2.15 Cell transfection

For siRNA or plasmid transfection, NP cells were seeded in 6−well plates at 3×10^5^ cells per well one day in advance. Transfection was performed when cells reached approximately 80% confluence. For complex preparation, 5 μL of Lipofectamine 2000 was diluted in 125 μL of serum−free DMEM−H medium (Tube I) and incubated at room temperature for 5 min. Separately, 50 nM siRNA was diluted in 125 μL of serum−free DMEM−H medium (Tube II). The two solutions were combined, gently mixed, and further incubated for 20 min at room temperature to allow complex formation. Subsequently, the medium in each well was replaced with 1.74 mL of serum−free DMEM−H, followed by dropwise addition of the transfection complexes. After gentle swirling of the plate, cells were incubated for 4–6 h before the medium was replaced with complete culture medium.

### 2.16 NO metabolites determination

Total nitric oxide (NO) metabolites (NO_3_^-^ + NO_2_^-^) in NP cell culture supernatants were quantified using a commercial assay kit (Jiancheng, Nanjing, China) according to the manufacturer’s instructions. Briefly, samples were incubated with the reagent mixture (R1 + R2) at 37°C for 60 min to reduce nitrate to nitrite. After addition of reagents R3 and R4, the tubes were vortexed, incubated for 40 min, and centrifuged at 4,000×g for 10 min. The resulting supernatant was then mixed with a freshly prepared chromogenic agent (R5 + R6 + R7) for 10 min. Absorbance was measured at 550 nm, and NO levels were expressed as fold changes relative to the control group.

### 2.17 RT-qPCR analysis

Total RNA was extracted using the AG RNAex Pro Reagent Kit (AG, Changsha, China). cDNA synthesis was performed with the Uni All-in-One First-Strand cDNA Synthesis SuperMix for qPCR (TransGen Biotech, Beijing, China). PCR amplification was conducted using the Green qPCR SuperMix Kit (TransGen Biotech, Beijing, China). Relative mRNA expression levels were calculated via the 2-ΔΔCt method, with *GAPDH* serving as the internal control. Primer sequences are listed in **Supplementary Table 3**.

### 2.18 Western blot

For western blot analysis, NP cells were lysed in RIPA buffer (Beyotime, Shanghai, China) supplemented with protease inhibitors (Roche, Basel, Switzerland). The lysate was centrifuged to isolate proteins, which were then quantified using a BCA assay kit (Solarbio, Beijing, China). Equal amounts of protein were separated by SDS-PAGE and transferred to PVDF membranes. Membranes were blocked for 2 h at room temperature and incubated overnight at 4°C with primary antibodies. After washing, membranes were treated with secondary antibodies for 1 h at room temperature. Protein bands were visualized using an ECL detection solution (Meilunbio, Dalian, China) and imaged with a Tanon-5200 system. Antibody details are provided in **Supplementary Table 4**.

### 2.19 Establishment of the IDD rat model

A total of 25 healthy male Sprague-Dawley rats (200–250 g, 12 weeks old) were randomly divided into five groups (n=5) (1): Sham group: no puncture or other treatments (2); IDD group: puncture and intragastric administration of equal amounts of saline every other day (3); L-QUE group: puncture and intragastric administration of 25 mg/kg QUE every other day (4); M-QUE group: puncture and intragastric administration of 50 mg/kg QUE every other day (5); H-QUE group: puncture and intragastric administration of 100 mg/kg QUE every other day.

An IDD rat model was established by puncturing the Co7–8 intervertebral space with a 20G needle (42). The procedure was as follows: Rats were anesthetized intraperitoneally with pentobarbital (40 mg/kg) and then placed in a prone position on a platform. The selected Co7–8 segments were confirmed using manual palpation and X-ray images. After disinfection with iodine, a 20G needle was vertically inserted through the skin into the NP tissue of the caudal vertebra, with a puncture depth of about 4 mm, confirmed by X-ray images. The needle was rotated 360 degrees for 45 s. Four weeks later, the Co7–8 IVD was harvested and studied. All experiments were conducted under sterile conditions. The rats were transferred to a warm, ventilated environment until anesthesia recovery. Then, the rats had unrestricted access to the same specific pathogen-free (SPF) standard food and water. The animal study protocol was approved by the Ethics Committee of the Second Affiliated Hospital of Harbin Medical University (No. YJSDW2024-111).

### 2.20 X-ray and magnetic resonance imaging

Four weeks post-operation, the rats were anesthetized and positioned in a supine manner with their tails extended. X-ray imaging and a MRI system (9.4T BioSpec MRI, Bruker, Germany) were used to assess the caudal vertebrae. Sagittal T2-weighted images were obtained with the following parameters: TR = 5000 ms, TE = 30 ms, FOV = 225 mm, matrix size = 150 × 150, and slice thickness = 0.5 mm.

### 2.21 Histological assessment

Four weeks post-surgery, rat tails were collected. After being washed with PBS, the tissues were fixed in 4% paraformaldehyde for 12 h. Subsequently, the samples were decalcified with 10% formic acid, gradually dehydrated with ethanol, and finally embedded in paraffin. The tissue sections were 5 μm thick. After deparaffinization and rehydration, the sections were stained with hematoxylin and eosin (HE), Safranin O-fast green (SO), and Alcian blue. Subsequently, the degree of IDD was quantified using a histological grading system, with scores ranging from 5 to 15 points (43).

### 2.22 Statistical analysis

Statistical analyses were performed using GraphPad Prism 9.0. Data are presented as mean ± standard deviation (SD). The normality of data distribution was evaluated using the Shapiro-Wilk test, and variance homogeneity was confirmed through the Brown-Forsythe test. Comparisons among multiple groups were conducted using one-way analysis of variance (ANOVA) followed by Tukey’s *post hoc* test. All experiments were performed in triplicate. Statistical significance was defined as ^*^P < 0.05, ^**^P < 0.01, and ^***^P < 0.001.

## 3 Results

### 3.1 Screening for potential targets

**Figure 1:**
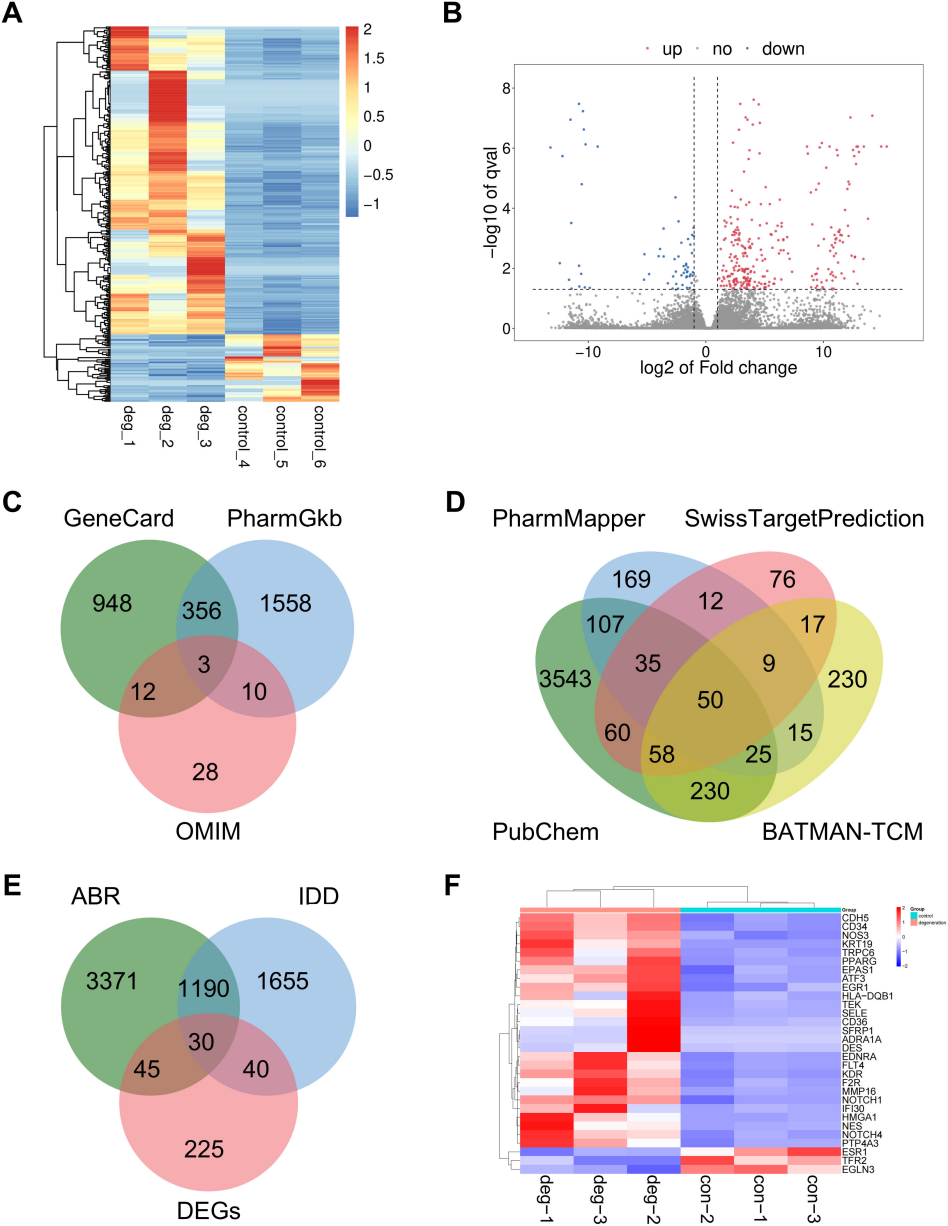
Screening for potential targets. **(A, B)** DEGs were obtained using RNA-seq based on control and degenerated human NP tissue (n = 3 per group). **(C)** IDD targets were sourced from GeneCard, PharmGkb, and OMIM. **(D)** ABR main compound targets were obtained from PubChem, PharmMapper, SwissTargetPrediction, and BATMAN-TCM. **(E)** By intersecting DEGs, IDD disease targets, and ABR main compound targets, we identified 30 overlapping targets. **(F)** The expression of 30 overlapping targets in IDD and control tissues (n = 3 per group).

Our investigation began with an integrated, multi-source screening to identify potential targets underlying ABR’s pharmacological action against IDD. RNA-seq analysis identified 340 DEGs in degenerated human NP tissue compared to controls (**Figures 1A, B**). Using GeneCards, PharmGkb, and OMIM databases, we retrieved 1,319, 1,927, and 53 IDD-related genes, respectively. After removing duplicates, 2,915 unique IDD-associated targets were obtained (**Figure 1C**) (**Supplementary Table 5**). From the TCMSP database, 176 ABR compounds were identified, with 21 meeting the criteria of OB ≥ 30% and DL ≥ 0.18 (**Supplementary Table 6**). The PubChem, PharmMapper, SwissTargetPrediction, and BATMAN-TCM databases yielded 4,108, 422, 317, and 634 predicted targets, respectively, resulting in 4,636 unique ABR-associated targets after deduplication (**Figure 1D**) (**Supplementary Table 7**). By intersecting DEGs, IDD-associated targets, and ABR-related targets, we identified 30 overlapping genes (**Figure 1E**). Expression analysis confirmed that these genes effectively distinguished IDD from control samples (**Figure 1F**).

### 3.2 Functional enrichment analysis

**Figure 2:**
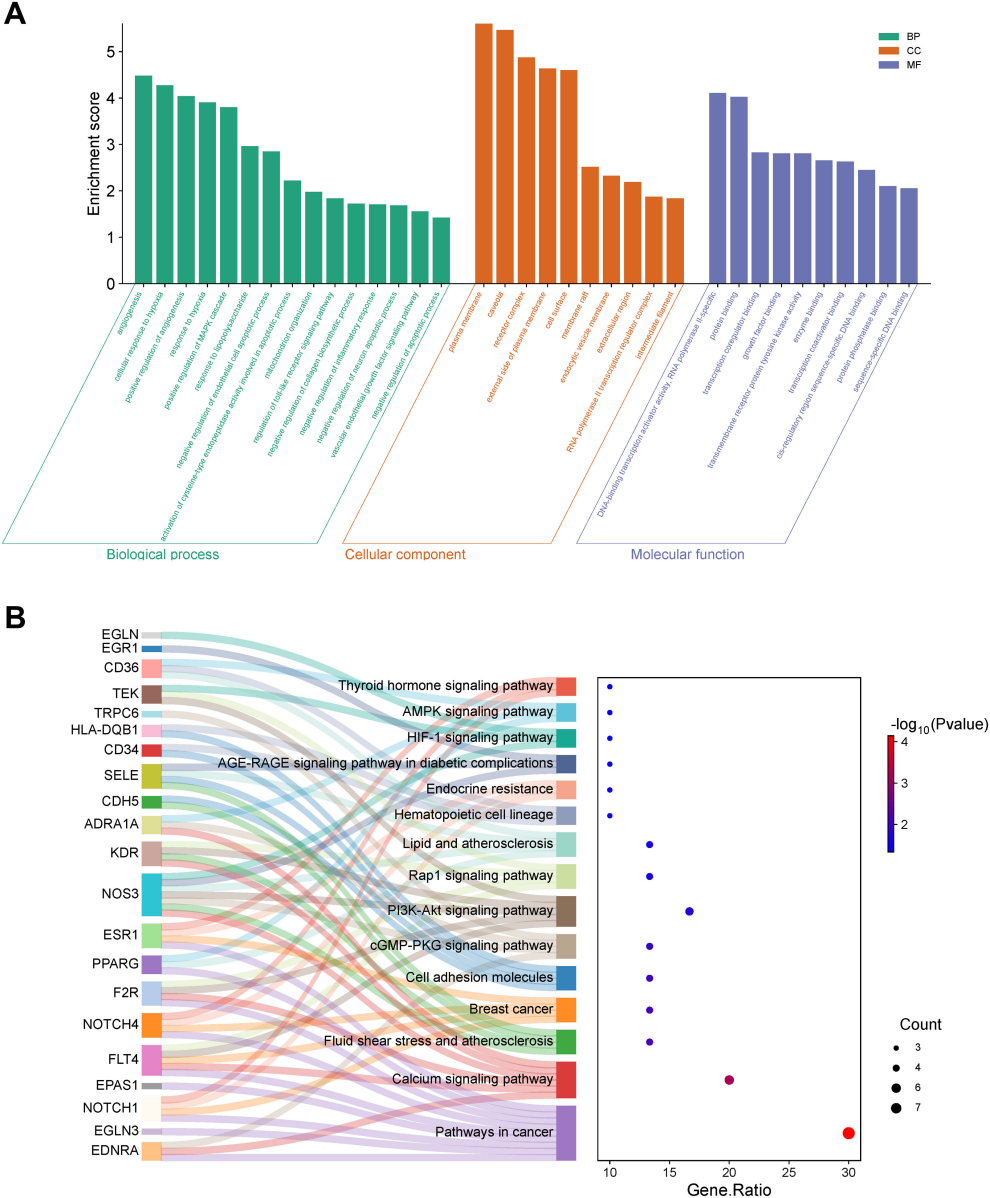
Functional enrichment analysis. **(A)** GO enrichment analysis. **(B)** Sankey and dot plot of gene-KEGG pathway.

To decipher the biological functions and pathways associated with the 30 overlapping genes, GO and KEGG pathway enrichment analyses were conducted using the DAVID database (*P* < 0.05) (**Supplementary Table 8**). GO analysis identified 111 biological process (BP) terms, including responses to hypoxia, mitochondrion organization, negative regulation of collagen biosynthesis, inflammatory response, apoptosis, and angiogenesis. Additionally, 17 cellular component (CC) terms were identified, primarily involving the plasma membrane, caveola, and receptor complexes, alongside 17 molecular function (MF) terms associated with protein binding, protein phosphatase binding, and signaling receptor activity. The GO analysis results are depicted in a bar chart (**Figure 2A**). KEGG pathway analysis revealed 15 IDD-related pathways, notably the HIF-1, Calcium, AMPK, PI3K-Akt, and AGE-RAGE signaling pathways (**Figure 2B**), suggesting that ABR may exert therapeutic effects in IDD by modulating these pathways.

### 3.3 Network analysis of common targets and compound-target-signaling network construction

**Figure 3:**
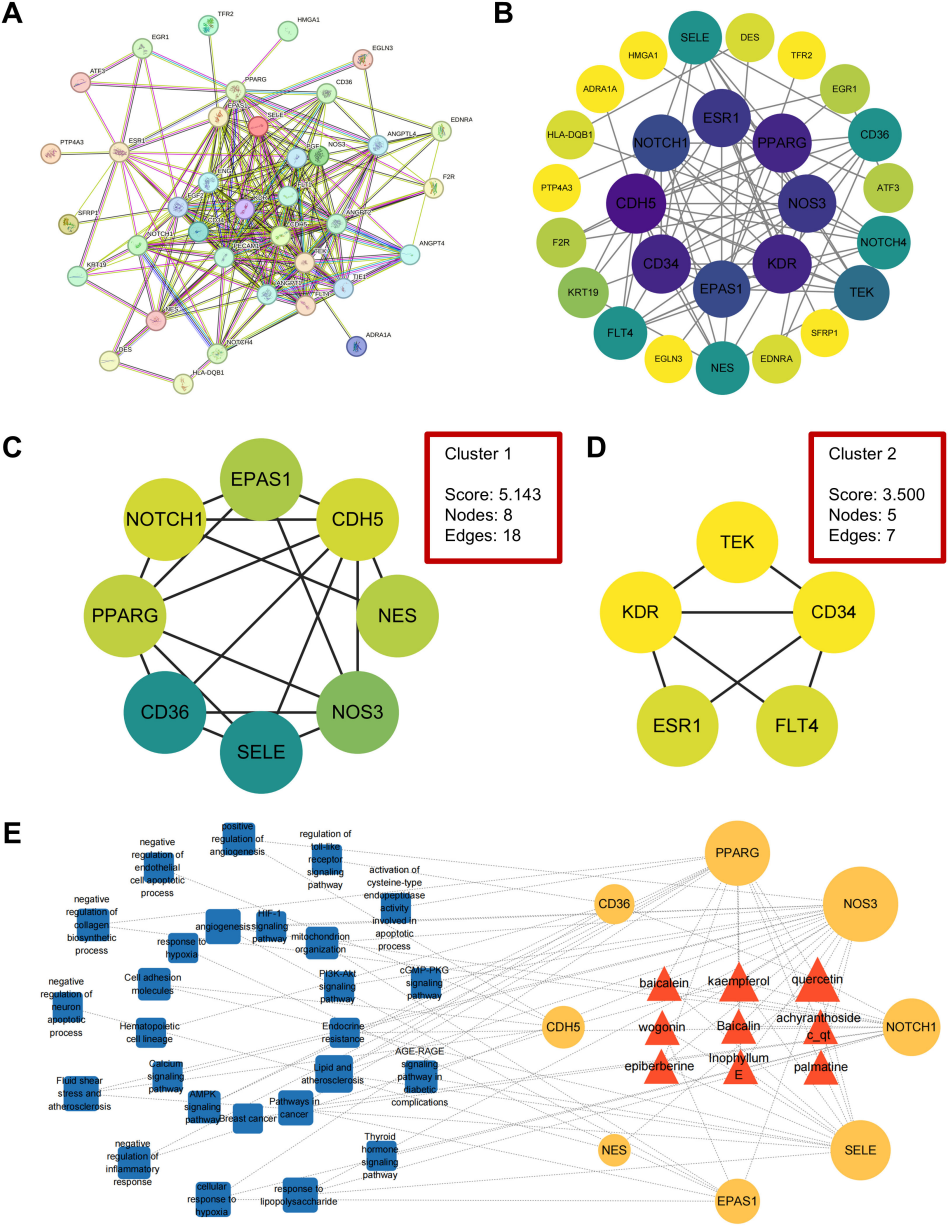
Network analysis of common targets and compound-target-signaling network construction. **(A)** The PPI network of 30 overlapping targets. **(B)** Visualization of the PPI network, with node color indicating the degree value. **(C, D)** The clustering graphs were obtained using the MCODE plugin. **(E)** The compound-target-signaling network was visualized with red triangles representing the primary drug components of ABR, orange circles indicating core targets, and blue squares denoting critical pathways, with node size representing degree value.

To further identify the core targets and explore their interactions with ABR components, we constructed a PPI network of the 30 overlapping genes using STRING (**Figure 3A**) and performed subsequent analysis in Cytoscape (**Figure 3B**). MCODE clustering analysis identified two key modular clusters (**Figures 3C, D**), with the highest-scoring cluster considered critical in ABR-mediated IDD treatment. This cluster highlighted eight core targets: *CDH5*, *PPARG*, *EPAS1*, *NOS3*, *NOTCH1*, *NES*, *CD36*, and *SELE*.

We employed Cytoscape software to construct the component-target-pathway network (**Figure 3E**). As shown, the top nine active compounds interacting with the eight hub genes included QUE, kaempferol, wogonin, baicalein, baicalin, epiberberine, palmatine, achyranthoside C_qt, and inophyllum E, collectively targeting 26 enriched pathways. Based on degree values, QUE and kaempferol exhibited the highest connectivity among ABR active compounds, interacting with seven and five genes, respectively, while each of the remaining seven compounds was linked to three genes. These findings position QUE and kaempferol as central compounds for further molecular docking and MD simulation analyses.

### 3.4 Molecular docking analysis

**Figure 4:**
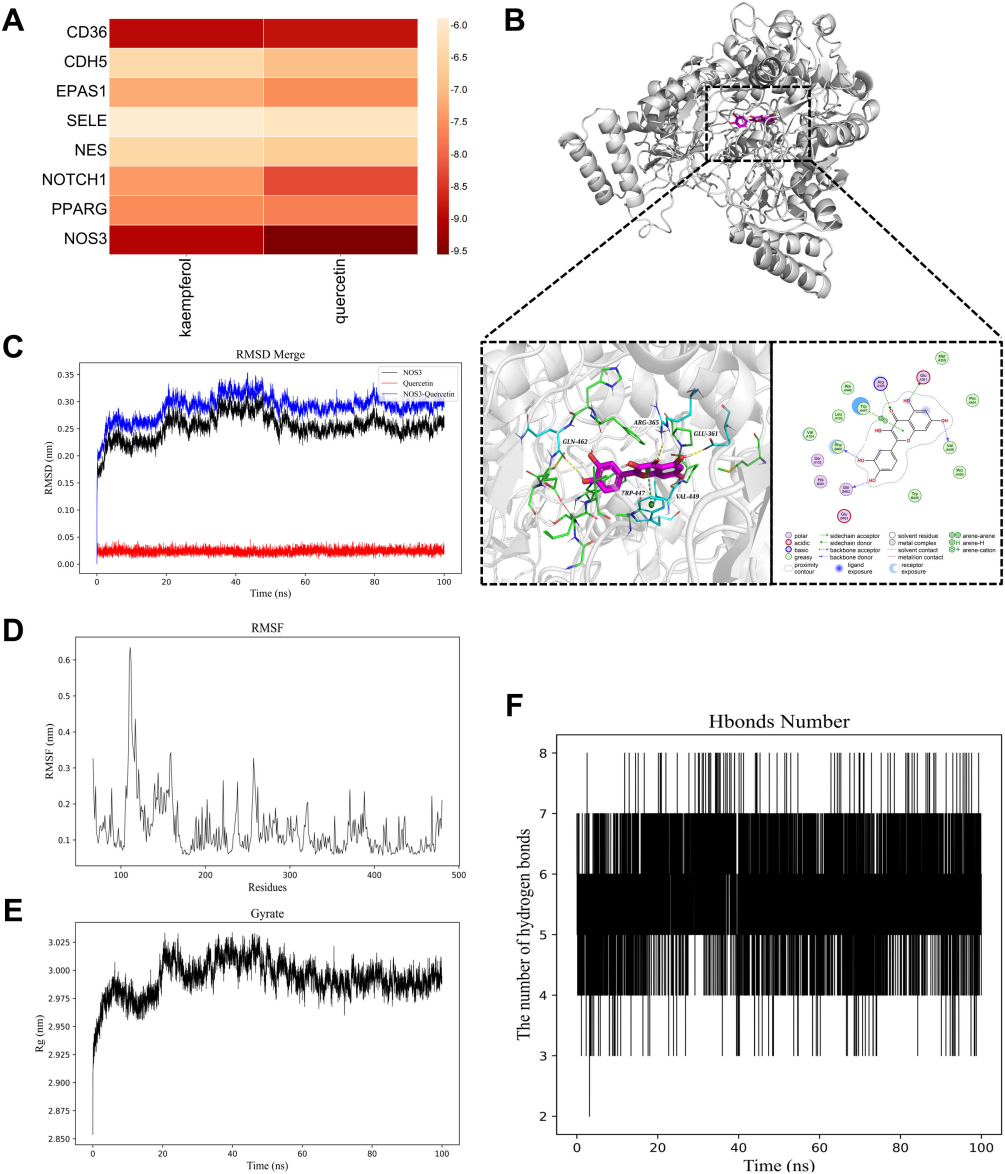
Molecular docking and MD simulation analysis. **(A)** Heatmap of molecular docking binding energy. **(B)** The 2D and 3D images show the combination of QUE and *NOS3*. **(C)** RMSD merge plot. **(D)** RMSF plot. **(E)** Rg plot. **(F)** Hydrogen bond analysis plot.

To validate the binding affinity of the key ABR components with central targets, molecular docking analysis was performed using AutoDock Vina (**Figure 4A**). In this study, all active compounds exhibited binding affinities below −5.0 kcal/mol, demonstrating favorable interactions with core targets. Notably, QUE displayed the highest binding affinity to *NOS3*, as reflected by its most negative docking score.

Further protein-ligand interaction analysis using PLIP (**Figure 4B**) revealed that QUE formed hydrogen bonds with *NOS3* at ARG 365A (2), VAL 449A, and GLN 462B. Additionally, hydrophobic interactions were observed with TRP 445B, ALA 446A, and PHE 460B, while π-stacking interactions with TRP 447A (2) further enhanced complex stability.

### 3.5 MD simulation analysis

Given its strongest binding affinity, the *NOS3*-QUE complex was selected for MD simulations to assess its structural stability and dynamic behavior. Key analyses included the root mean square deviation (RMSD) plot, root mean square fluctuation (RMSF) plot, radius of gyration (Rg) plot, and hydrogen bond analysis plot.

The RMSD of the *NOS3*-QUE complex stabilized at 0.30–0.35 nm after 20 ns, indicating structural integrity (**Figure 4C**). RMSF values indicated moderate overall flexibility, with residues 100–150 showing elevated fluctuations (**Figure 4D**). Rg values stabilized within 2.95–3.02 nm after an initial decline, reinforcing structural compactness (**Figure 4E**). Hydrogen bond numbers fluctuated dynamically but maintained an average of 5–6 bonds, confirming stable intermolecular interaction (**Figure 4F**). Collectively, the *NOS3*−QUE complex demonstrates robust structural stability, functionally relevant flexibility, and persistent hydrogen−bond interactions that support its biological binding mode.

These in silico findings provided a clear rationale for subsequent experiments. Based on network and molecular docking results, QUE was prioritized as the core ABR component due to its highest connectivity and strongest predicted binding to *NOS3*. This selection is further supported by recent UPLC−MS/MS analyses, which confirm QUE as a principal flavonoid constituent of ABR (16). The predicted QUE-*NOS3* interaction was further validated by stable MD simulation analysis. Pathway enrichment further revealed the PI3K-Akt axis as significantly implicated, with *NOS3* concurrently identified as a key network hub and known downstream effector of this pathway. The convergence of these predictions led to a testable hypothesis: QUE alleviates IDD by modulating the PI3K/Akt/eNOS axis. This hypothesis directly guided all subsequent *in vitro* and *in vivo* experimental designs.

### 3.6 QUE alleviates IL-1β-induced NP cell dysfunction

**Figure 5:**
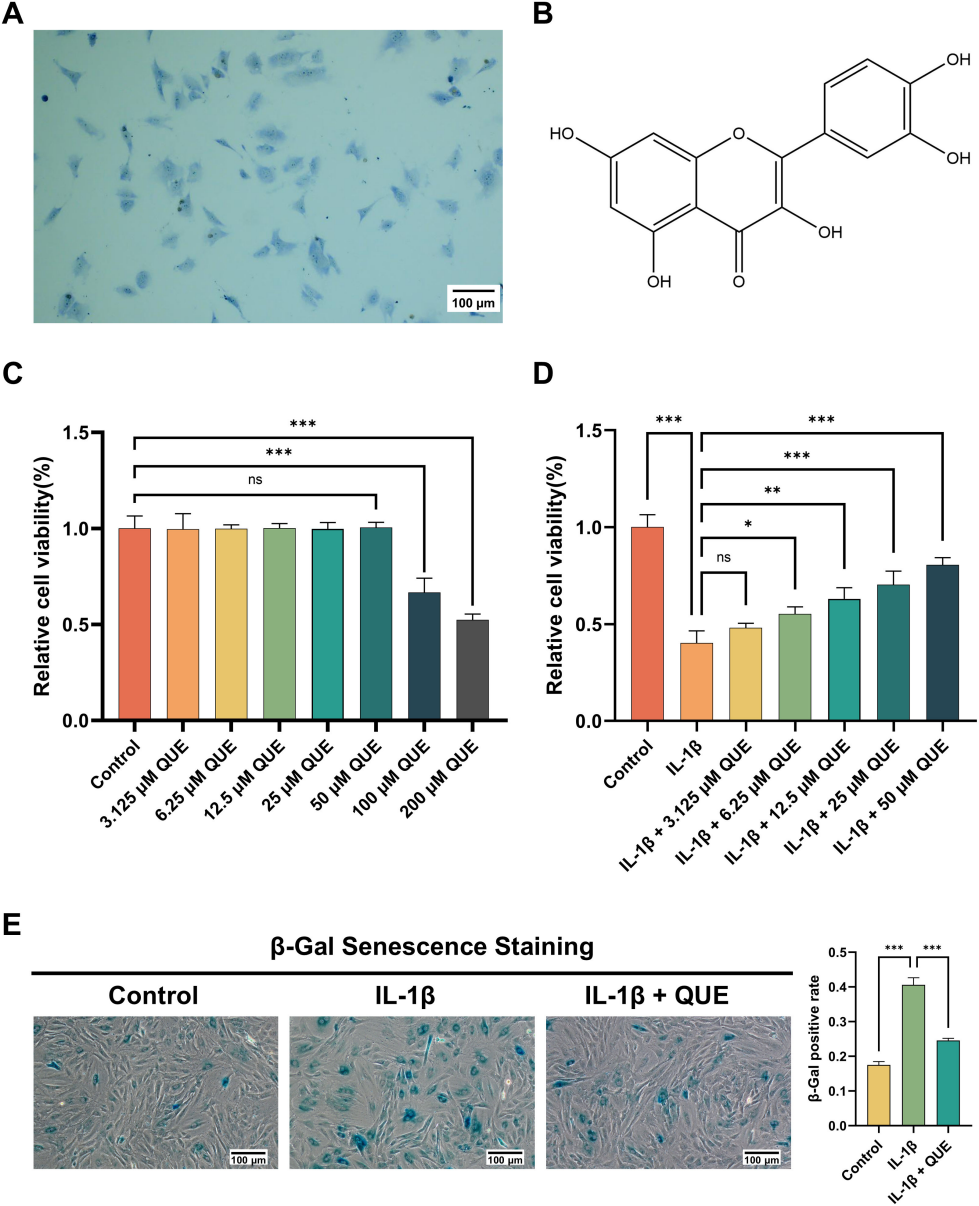
QUE alleviates IL-1β-induced NP cell dysfunction. **(A)** Toluidine blue staining was used to identify NP cells. Scale bars = 100 μm. **(B)** The chemical structure of QUE. **(C)** NP cells were exposed to varying concentrations of QUE (3.125, 6.25, 12.5, 25, 50, 100, and 200 μM), and cell viability was subsequently evaluated (n = 3). **(D)** NP cells exposed to IL-1β treatment (20 ng/mL for 48 h) were subjected to gradient concentrations of QUE (3.125, 6.25, 12.5, 25, and 50 μM), and cell viability was evaluated (n = 3). **(E)** β-Gal senescence staining was conducted to assess NP cell senescence (n = 3). Scale bars = 100 μm. Data are presented as mean ± SD. Statistical analysis was performed using one-way ANOVA followed by Tukey’s *post hoc* test. ^ns^*P* > 0.05; ^*^P < 0.05; ^**^P < 0.01; ^***^P < 0.001.

To identify NP cells, toluidine blue staining was performed (**Figure 5A**). We then focused on QUE, the key ABR component identified in our network analysis (**Figure 5B**), which possesses well-documented antioxidant and anti-inflammatory properties (44). To assess its cytotoxicity, NP cells were treated with QUE at concentrations ranging from 3.125 to 200 μM for 48 h, followed by viability analysis (**Figure 5C**). QUE at 3.125–50 μM showed no significant cytotoxicity, whereas 100 and 200 μM markedly reduced cell viability. Therefore, 3.125–50 μM QUE was deemed safe for further experiments. To determine the optimal QUE concentration, NP cells pretreated with IL-1β (20 ng/mL, 48 h) were exposed to increasing QUE concentrations (3.125–50 μM), followed by viability assessment (**Figure 5D**). While 3.125 μM QUE had minimal protective effects, 6.25–50 μM partially restored IL-1β-induced cell viability loss, with 50 μM demonstrating the most significant benefit. Consequently, 50 μM QUE was selected for subsequent experiments. Furthermore, β-Gal staining confirmed that IL-1β significantly increased senescent cell numbers, whereas QUE treatment partially reversed IL-1β-induced senescence (**Figure 5E**). Collectively, these initial experiments confirmed that QUE safely and effectively ameliorates IL-1β-induced NP cell dysfunction and senescence, supporting its functional relevance as a core ABR constituent against IDD.

### 3.7 QUE alleviates oxidative damage, ECM degradation, and inflammatory responses in NP cells induced by IL-1β

**Figure 6:**
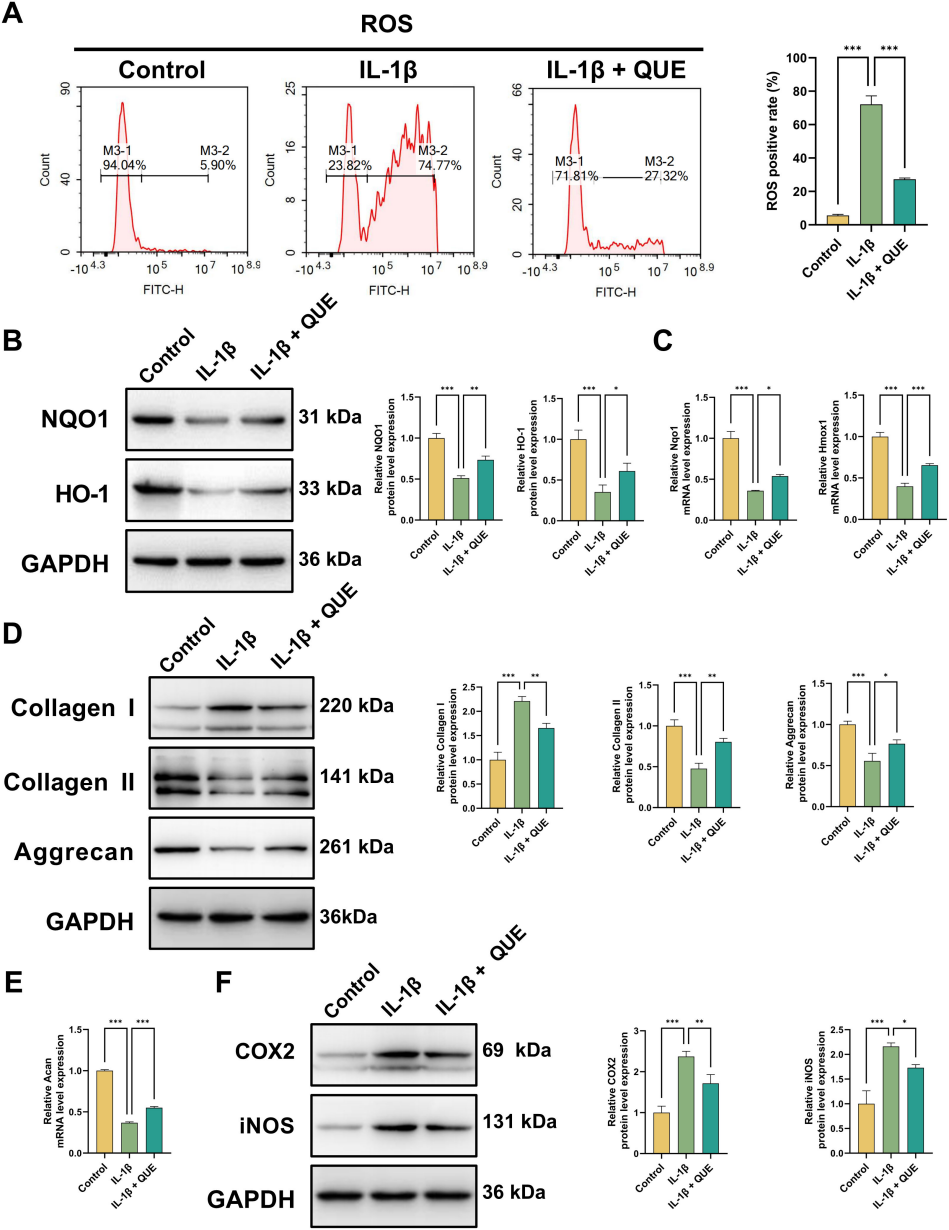
QUE alleviates NP cell oxidative damage, ECM degradation, and inflammatory responses induced by IL-1β. **(A)** ROS measurement and quantitative analysis of NP cells (n = 3). **(B)** WB analysis was employed to examine the NQO1 and HO-1 protein levels (n = 3). **(C)** Relative mRNA expression of *Nqo1* and *Hmox1* (n = 3). **(D)** WB analysis was employed to examine the Aggrecan, Collagen I, and Collagen II protein levels (n = 3). **(E)** Relative mRNA expression of *Acan* (n = 3). **(F)** WB analysis was employed to examine the COX2 and iNOS protein levels (n = 3). Data are presented as mean ± SD. Statistical analysis was performed using one-way ANOVA followed by Tukey’s *post hoc* test. ^*^P < 0.05; ^**^P < 0.01; ^***^P < 0.001.

Having established that QUE alleviates cellular dysfunction, we next aimed to elucidate its specific effects on the core pathological processes of IDD. Excessive ROS play a key role in IDD by promoting ECM degradation and inflammation (45). Consistently, flow cytometry revealed a significant IL-1β-induced ROS increase in NP cells, which QUE treatment effectively suppressed (**Figure 6A**). Western blot analysis confirmed that IL-1β downregulated antioxidant proteins NQO1 and HO-1, while QUE restored their expression (**Figure 6B**). RT-qPCR further validated that QUE mitigated the IL-1β-induced suppression of *Nqo1* and *Hmox1* mRNA levels (**Figure 6C**). Next, the impact of QUE on IL-1β-driven ECM degradation was investigated. Western blot showed that IL-1β reduced Aggrecan and Collagen II expression while increasing Collagen I levels, whereas QUE counteracted these effects (**Figure 6D**). RT-qPCR analysis of *Acan* expression confirmed these findings (**Figure 6E**). Finally, to assess QUE’s anti-inflammatory potential in IDD, we analyzed the key proinflammatory markers COX2 and iNOS. Western blot revealed that QUE partially inhibited IL-1β-induced COX2 and iNOS upregulation (**Figure 6F**). In summary, these results demonstrate that QUE comprehensively mitigates IL-1β-induced oxidative stress, ECM degradation, and inflammation in NP cells.

### 3.8 QUE alleviates IL-1β-caused apoptosis

**Figure 7:**
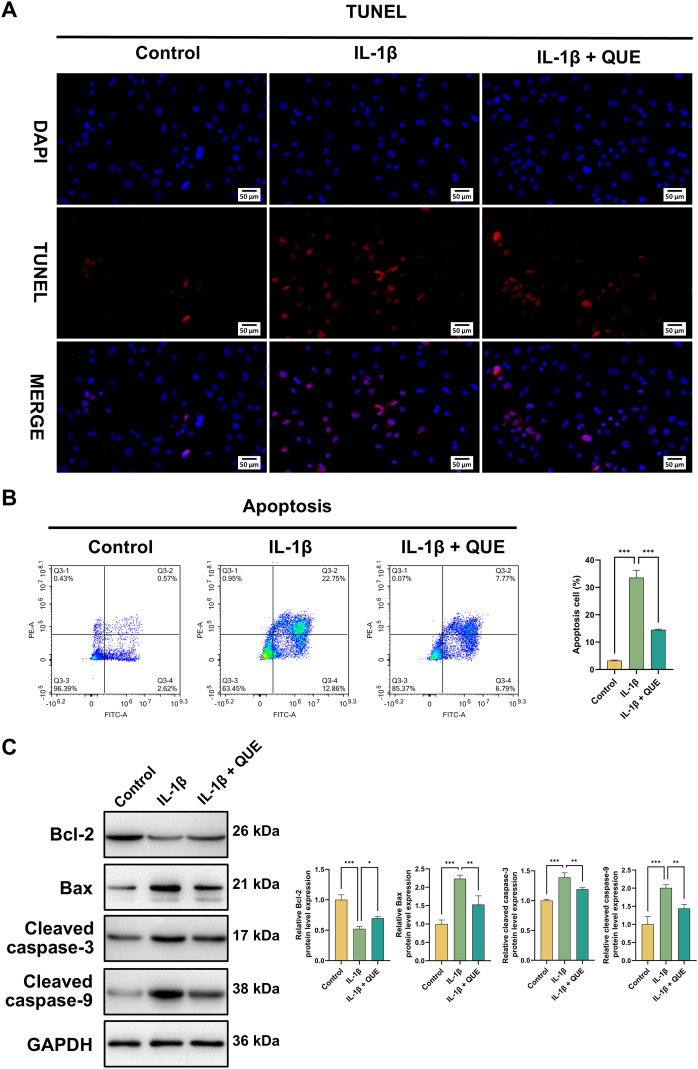
QUE alleviates IL-1β-caused apoptosis and senescence. **(A)** TUNEL assays were conducted to assess apoptosis in NP cells. Scale bars = 50 μm (n = 3). **(B)** Flow cytometry and quantitative analysis of NP cell apoptosis. Apoptotic cells were defined as cells that were positive for FITC Annexin V but negative for PI, along with those positive for both markers (n = 3). **(C)** WB analysis was employed to examine the Bcl-2, Bax, cleaved caspase-3, and cleaved caspase-9 protein levels (n = 3). Data are presented as mean ± SD. Statistical analysis was performed using one-way ANOVA followed by Tukey’s *post hoc* test. ^*^P < 0.05; ^**^P < 0.01; ^***^P < 0.001.

Based on the recognized association between apoptosis and IDD (46), we next investigated whether QUE could protect NP cells from IL-1β-induced apoptosis. TUNEL staining indicated that IL-1β significantly increased apoptotic cells, with QUE reversing this effect (**Figure 7A**). Flow cytometry further confirmed that QUE markedly reduced IL-1β-induced apoptosis (**Figure 7B**). Additionally, western blot analysis showed that IL-1β upregulated the pro−apoptotic proteins Bax, cleaved caspase-3, and cleaved caspase-9, while downregulating the anti−apoptotic protein Bcl-2; these effects were reversed by QUE treatment (**Figure 7C**). These results collectively demonstrate that QUE protects NP cells from IL-1β-induced apoptosis.

### 3.9 Effects of QUE on the IL-1β-caused PI3K/Akt/eNOS pathway in NP cells

**Figure 8:**
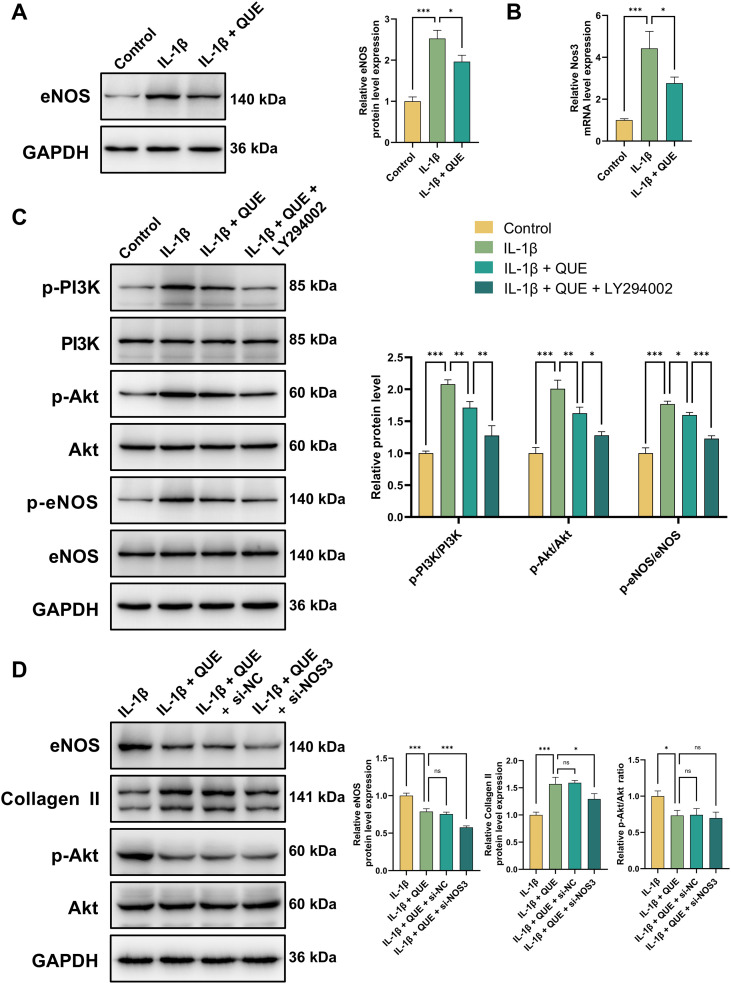
Effects of QUE on the IL-1β-caused PI3K/Akt/eNOS pathway in NP cells. **(A)** WB analysis was employed to examine the eNOS protein levels (n = 3). **(B)** Relative mRNA expression of *Nos3* (n = 3). **(C)** WB analysis was employed to examine the PI3K/Akt/eNOS pathway protein levels (n = 3). **(D)** Western blot analysis of eNOS, Collagen II, p-AKT, and AKT protein levels (n = 3). Data are presented as mean ± SD. Statistical analysis was performed using one-way ANOVA followed by Tukey’s *post hoc* test. *^ns^P* > 0.05; ^*^P < 0.05; ^**^P < 0.01; ^***^P < 0.001.

Using network pharmacology and RNA-seq, we identified *NOS3* as a potential core gene among ABR-predicted targets for IDD. To validate its role, we examined *Nos3* expression in IL-1β-induced NP cells. RT-qPCR and Western blot analyses confirmed that QUE attenuated the IL-1β-induced upregulation of *Nos3* mRNA and eNOS protein levels in NP cells (**Figures 8A, B**).

Enrichment analysis revealed that the 30 overlapping genes were associated with key signaling pathways, including HIF-1, Calcium, AMPK, PI3K-Akt, and AGE-RAGE. The PI3K-Akt pathway has been widely recognized as a promising therapeutic target for IDD (47, 48). Given that eNOS is a crucial downstream effector of the PI3K-Akt pathway (49), we investigated its involvement in QUE-mediated IL-1β-induced IDD treatment. In IL-1β-induced NP cells, the phosphorylation ratios of p-PI3K/PI3K, p-Akt/Akt, and p-eNOS/eNOS were significantly increased, indicating PI3K/Akt/eNOS pathway activation (**Figure 8C**). However, QUE effectively suppressed the IL-1β-induced phosphorylation of PI3K, Akt, and eNOS, demonstrating its therapeutic effect. Additionally, the PI3K inhibitor LY294002 further reduced phosphorylation levels, confirming eNOS as a downstream target of the PI3K-Akt pathway in QUE-mediated IDD treatment. Collectively, our findings suggest that QUE exerts therapeutic effects in IL-1β-induced NP cells by modulating the PI3K/Akt/eNOS pathway.

To further establish the functional necessity of *Nos3*within this pathway, we performed siRNA-mediated knockdown. Western blot analysis confirmed efficient eNOS depletion (**Figure 8D**). Notably, QUE retained its ability to suppress Akt phosphorylation even under *Nos3* knockdown conditions. In contrast, the protective effect of QUE on the synthesis of Collagen II—a key ECM component—was significantly attenuated upon *Nos3* knockdown. This demonstrates that while QUE’s inhibition of Akt phosphorylation occurs upstream of eNOS, its promotion of matrix synthesis is critically dependent on eNOS.

To functionally assess the PI3K/Akt/eNOS pathway, total NO metabolites (NO_3_^-^ + NO_2_^-^) in NP cell supernatants were measured (**Supplementary Figure 2**). Consistent with the increased phosphorylation of Akt and eNOS observed in **Figure 8C**, IL-1β stimulation significantly elevated NO production, confirming pathway activation. QUE partially reversed IL-1β-induced NO overproduction, whereas the PI3K inhibitor LY294002 almost completely abolished it. Co-treatment with QUE and LY294002 did not further reduce NO levels, indicating that QUE acts upstream within the PI3K-Akt pathway. *Nos3* knockdown abolished the IL−1β−induced NO elevation, and QUE exerted no additional inhibitory effect under *Nos3*−deficient conditions, demonstrating that QUE’s suppression of NO production is dependent on eNOS. Collectively, these results confirm that QUE inhibits IL-1β-induced NO production via the PI3K/Akt/eNOS pathway, with eNOS serving as a critical downstream effector.

Collectively, these findings indicate that QUE exerts its therapeutic effects in IL-1β-induced NP cells by modulating the PI3K/Akt/eNOS pathway, with *Nos3* serving as an indispensable downstream effector for ECM protection.

### 3.10 QUE attenuates IDD in rats

**Figure 9:**
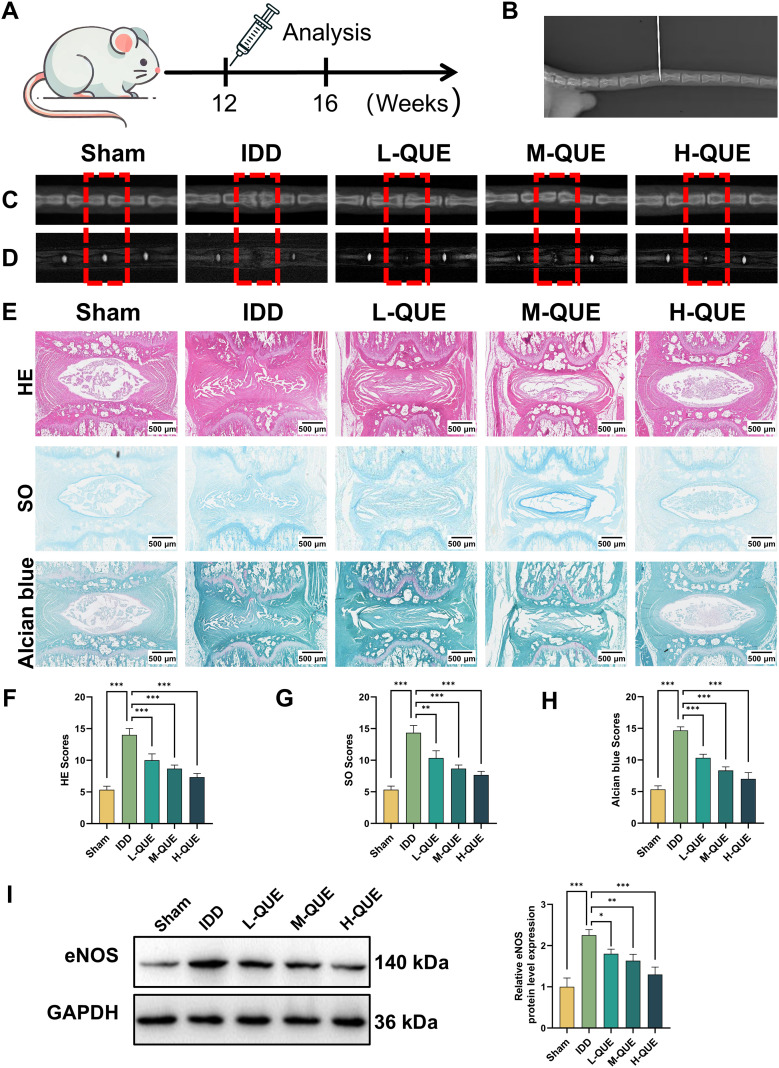
QUE attenuates IDD in rats. **(A)** Overview of animal experiments. **(B)** X-ray was used to confirm the establishment of the IDD model in rats. **(C)** Representative X-ray images of the caudal vertebrae of rats at 4 weeks post-operation (marked by red wireframe). **(D)** Representative MRI images of the caudal vertebrae of rats at 4 weeks post-operation (marked by red wireframe). **(E–H)** The HE, SO, Alcian blue staining images of rat IVDs in each group at 4 weeks post-operation, scale bar = 500 μm. Histological score of each group was calculated (n = 5). **(I)** WB analysis was employed to examine the eNOS protein levels (n = 3). Data are presented as mean ± SD. Statistical analysis was performed using one-way ANOVA followed by Tukey’s *post hoc* test. ^*^P < 0.05; ^**^P < 0.01; ^***^P < 0.001.

*In vitro* experiments have demonstrated the significant therapeutic effect of QUE, a primary active component of ABR, on IDD. This study further established the IDD animal model for *in vivo* experiments to verify the therapeutic effect of QUE (**Figure 9A**). Manual palpation and X-ray were used to confirm the selected Co7–8 segments for the puncture operation (**Figure 9B**).

The X-ray findings indicated that, compared to the sham group, the IDD group exhibited significant disc space narrowing after puncture (**Figure 9C**). The QUE treatment groups showed significant improvement in disc height, with the H-QUE group showing the best therapeutic effect. The MRI findings revealed a significantly lower signal intensity in the IDD group compared to the sham group (**Figure 9D**). After QUE treatment, the NP signal intensity significantly increased, with the H-QUE group showing the most pronounced effect. Overall, the X-ray and MRI results were consistent, indicating that QUE significantly improved IDD in rats.

HE staining revealed that the NP structure exhibited atrophy and was replaced by proliferative connective tissue, with rupture of the AF and severe disruption of the boundary between the NP and AF in the IDD group (**Figures 9E, F**). However, after 4 weeks of QUE treatment, significant dose-dependent improvements in IVD structure and morphology were observed, including notable repair of NP, improved AF appearance, clearer boundary between NP and AF, and significant restoration of IVD height. SO and Alcian Blue staining further corroborated the HE staining results (**Figures 9G, H**).

Finally, western blot revealed that the eNOS expression was significantly higher in the IDD group compared to the sham group. In contrast, QUE treatment groups significantly reduced eNOS expression, with the H-QUE group showing the most pronounced therapeutic effect (**Figure 9I**).

## 4 Discussion

IDD is a chronic, progressive, and multifactorial disorder that ranks among the leading causes of chronic LBP (50). In the clinical practice, ABR is a commonly used Chinese medicinal herb in traditional compound prescriptions (12), yet the precise mechanisms of its action in IDD treatment remain unclear. To bridge this gap, we applied network pharmacology, an emerging approach that integrates big data analysis, to elucidate the therapeutic mechanisms in this study (51). Our integrated analysis identified QUE as the central constituent of ABR. Consequently,we focused our experimental investigation on this key component to unravel ABR’s mechanism in IDD.

ABR exhibits a broad spectrum of biological activities, including neuroprotective, anti-inflammatory, and antioxidant effects (18, 52). Its active constituents contribute to protective effects across multiple organ systems, such as the heart, bones, liver, kidneys, eyes, and nervous system (14, 53). Notably, ABR is frequently incorporated into traditional formulations for IDD treatment, where it has been shown to mitigate oxidative damage, enhance ECM synthesis, and promote NP cell proliferation (12, 54). Network pharmacology analysis using the TCMSP database identified 21 primary active compounds in ABR that met the criteria of OB ≥ 30% and DL ≥ 0.18. Component-target-pathway analysis revealed nine key bioactive compounds: quercetin (QUE), kaempferol, wogonin, baicalein, baicalin, epiberberine, palmatine, achyranthoside C_qt, and inophyllum E. Consistent with modern phytochemical characterization, QUE has been identified as a major flavonoid constituent of ABR (16, 17). Building on this foundation, our network pharmacology results further confirmed its key role against IDD, supporting its selection as the focus for mechanistic investigation. Studies have demonstrated that QUE protects against IDD via the Keap1/Nrf2 and SIRT1-autophagy pathways (55). Kaempferol enhances NP cell viability, inhibits apoptosis and senescence, and promotes ECM synthesis, likely via the MAPK signaling pathway (8). Wogonin alleviates IDD-related pain by downregulating *NGF* expression in intervertebral discs (56). Baicalein suppresses inflammation and ECM degradation in IDD (57), while baicalin reduces apoptosis, oxidative stress, inflammation, and ECM breakdown in human NP cells (58). Epiberberine exhibits anti-inflammatory, anti-hyperlipidemic, and anti-hyperglycemic properties and shows therapeutic potential for osteolytic damage caused by breast cancer (59). Palmatine has demonstrated anticancer, antioxidant, and anti-inflammatory effects, with potential applications in osteoarthritis treatment (60). However, no direct studies have investigated the roles of achyranthoside C_qt and inophyllum E in bone-related disorders. This study establishes QUE as a primary active component of ABR, providing a crucial pharmacological foundation for the future development and application of ABR in IDD therapy.

RNA-seq and bioinformatics analyses of IDD and control specimens identified 340 DEGs, comprising 279 upregulated and 61 downregulated genes. Further analysis identified 4,636 drug-related targets. Screening disease-related databases yielded 2,915 IDD-associated targets. By intersecting these datasets—2,915 disease targets, 340 DEGs, and 4,636 drug targets—30 overlapping hub genes were identified. PPI network construction and core target screening further refined this to eight key targets: *CDH5*, *NOS3*, *PPARG*, *EPAS1*, *NOTCH1*, *NES*, *CD36*, and *SELE*. *CDH5*, a transmembrane adherens protein, regulates endothelial permeability, angiogenesis, and vascular remodeling (61). *NOS3* (eNOS) produces NO, which modulates pain-related inflammation and vascular remodeling (62). In osteocytes, *PPARG* governs energy metabolism and bone physiology by regulating mitochondrial activity, ATP production, and ROS accumulation (63). *EPAS1* contributes to cartilage degradation by modulating matrix metalloproteinases and ADAMTS enzymes, playing a key role in ECM degradation and hypoxia responses in IDD (64). *NOTCH1* facilitates osteogenic differentiation and ECM mineralization in bone (65). *NES*, a marker for mesenchymal and hematopoietic stem cells, is expressed in osteoblasts, endothelial cells, and pericytes during skeletal development (66). *CD36* regulates angiogenesis, fatty acid metabolism, and inflammasome activation (67). *SELE* mediates the adhesion and homing of endothelial progenitor cells, supporting neovascularization (68).

Based on these enrichment analyses, we employed GO and KEGG analyses to further identify key biological processes and pathways implicated in the potential therapeutic mechanisms of ABR for IDD. These include hypoxia response (69), mitochondrial organization (70), negative regulation of collagen biosynthesis (71), suppression of inflammatory responses (72), apoptosis (8), and angiogenesis (73). Relevant signaling pathways include HIF-1 (74), calcium (75), AMPK (76), PI3K-Akt (77), and AGE-RAGE in diabetic complications (78), all of which have been implicated in IDD progression.

Following the initial computational screening, molecular docking experiments further confirmed interactions between key active compounds, QUE and kaempferol, with core targets. Molecular docking and MD simulations revealed that QUE, a primary ABR component, exhibits strong binding affinity for *NOS3* (eNOS). Notably, eNOS was significantly upregulated in IDD tissues. *In vitro*, IL-1β-stimulated NP cells showed increased eNOS and iNOS expression, which QUE treatment effectively reversed. NO, a short-lived signaling molecule synthesized by nitric oxide synthase (NOS), is widely recognized for its role as a physiological mediator (79). It regulates orthopedic processes, including inflammation, arthritis, and osteoporosis, and is linked to proteoglycan synthesis in intervertebral discs under varying hydrostatic pressures (80). Among NOS subtypes, eNOS and iNOS regulate bone remodeling and contribute to inflammatory responses, particularly in chronic pain syndromes (62). Our findings indicate that QUE alleviates IL-1β-induced upregulation of eNOS and iNOS. However, the precise mechanisms underlying eNOS, iNOS, and NO production in IDD pathogenesis remain incompletely understood.

We therefore experimentally verified the protective effect of QUE against multiple key pathological processes and cellular dysfunctions induced by IL-1β in NP cells. IL-1β plays a central role in NP cell suppression, oxidative stress, ECM degradation, inflammation, apoptosis, and cellular senescence—findings consistent with the GO enrichment analysis in this study (8, 46, 50). Oxidative stress is a key driver of IDD onset and progression, with excessive ROS accumulation triggering cellular damage and functional impairment (81). Increasing evidence suggests that Nrf2 pathway activation mitigates IDD by upregulating antioxidant gene expression, including *Nqo1* and *Hmox1* (82). The ECM metabolism within the inflammatory microenvironment plays a pivotal role in IDD pathogenesis. IDD is characterized by the progressive decline of matrix synthesis markers, such as Aggrecan and Collagen II, which are gradually replaced by Collagen I, leading to increased rigidity and fibrosis in the NP (83, 84). Additionally, inflammation is a critical driver of IDD, marked by significant infiltration of immune cells and elevated levels of pro-inflammatory mediators, including iNOS and COX2 (85, 86). Moreover, excessive apoptosis and senescence of NP cells compromise their normal function, making them potential therapeutic targets for IDD treatment (87). In this study, our *in vitro* experiments demonstrated that QUE effectively counteracted IL-1β-induced reductions in NP cell viability and attenuated oxidative stress, ECM degradation, inflammation, apoptosis, and cellular senescence. These findings provide deeper insights into the molecular mechanisms underlying the action of QUE, a key component of ABR, against IDD within our cellular model.

Several studies have identified key downstream effectors of the PI3K-Akt pathway, including MAPK, eNOS, mTOR, FoxO, and NF-κB, which regulate immune modulation, inflammation, cell proliferation, apoptosis, differentiation, and autophagy (47, 49). During IDD, the PI3K-Akt pathway plays a central role in apoptosis regulation, inflammatory responses, and ECM degradation, making it a promising therapeutic target (48). Notably, QUE has been shown to alleviate IDD through the Keap1/Nrf2 and SIRT1-autophagy pathways (55). However, its impact on the PI3K-Akt pathway and its downstream signaling in IDD progression remains unexplored. Given that eNOS functions as a key downstream effector of the PI3K-Akt pathway (49), this study investigated the role of the PI3K/Akt/eNOS pathway in mediating QUE’s therapeutic effects on IL-1β-induced cellular model. Our findings revealed increased phosphorylation of PI3K and Akt in IL-1β-stimulated NP cells, consistent with previous studies (50, 86, 88). Interestingly, IL-1β also promoted eNOS phosphorylation, which was reversed by QUE, highlighting its inhibitory effects on the PI3K/Akt/eNOS pathway in IL-1β-stimulated NP cells. To further validate these findings, we utilized the PI3K inhibitor LY294002, which suppressed the phosphorylation of PI3K, Akt, and eNOS, reinforcing the role of eNOS as a downstream effector in QUE-mediated IDD treatment. Consistently, *Nos3* knockdown abolished QUE’s protection of ECM synthesis while leaving its inhibition of Akt phosphorylation, establishing *Nos3* as an essential downstream component required for QUE’s therapeutic action via the PI3K-Akt pathway.

It is important to distinguish between the direct interaction of QUE with the eNOS protein and its effect on *NOS3* expression levels. Our MD simulations predict stable binding of QUE to *NOS3*, suggesting a direct, post-translational mechanism to modulate enzyme activity. Independently, the observed downregulation of IL-1β-induced *Nos3* mRNA and eNOS protein points to transcriptional or translational control. This is potentially mediated through QUE’s inhibition of the upstream PI3K-Akt pathway, a key regulator of *Nos3* expression. Supporting this, QUE has been shown in other systems to modulate eNOS levels via pathways such as SIRT1 and AMPK (89, 90). Thus, QUE appears to target the PI3K/Akt/eNOS axis through a dual strategy: suppressing the activating pathway to reduce eNOS synthesis and directly engaging the enzyme to constrain its activity.

Our results further confirm that IL-1β-induced NO overproduction in NP cells mainly depends on eNOS. QUE partially inhibits IL-1β-induced NO overproduction by regulating the PI3K/Akt/eNOS pathway, which is consistent with its ability to downregulate the expression of phosphorylated eNOS and *Nos3*. Experiments with LY294002 and si-NOS3 further verify that QUE exerts its regulatory effect by acting on the PI3K-Akt pathway and relies on the presence of eNOS. This finding further improves the protective mechanism of QUE against IDD and provides important pharmacological evidence for its potential clinical application in IDD treatment. These findings establish a novel understanding of the non-endothelial biology of *NOS3* (eNOS) in NP cells, thus highlighting its potential as a therapeutic target in IDD. Our results align with existing evidence that IDD is associated with multiple pathological processes, including apoptosis and neovascularization (85), as supported by GO analysis. The PI3K/Akt/eNOS pathway has been implicated in various physiological and pathological processes, particularly angiogenesis (91). Microvascular changes are considered a key factor in IDD progression, as microvascular invasion into NP cells and the CEP exacerbates degeneration (92). Consistently, our findings demonstrate that QUE inhibits the PI3K/Akt/eNOS pathway in IL-1β-induced NP cells. This mechanism may contribute to its protective effects against IDD progression, potentially by interfering with related neovascularization processes.

To further evaluate the therapeutic efficacy of QUE *in vivo*, we established a puncture-induced rat model of IDD for investigation. In this model, X-ray, MRI, and histological findings demonstrated that QUE could attenuate IDD progression. Moreover, we observed that QUE suppressed eNOS expression in the degenerative disc tissues. Collectively, our *in vivo* findings confirmed the therapeutic potential of QUE in the treatment of IDD.

In summary, our integrated approach establishes QUE as a pivotal component of ABR with therapeutic efficacy against IDD. Several notable limitations of this work must be considered. The IVD is an integrated structure composed of the NP, AF, and CEP, whereas our *in vitro* functional and mechanistic experiments here were confined to NP cells. In addition, due to limited experimental resources, a quantitative analysis of the specific content and proportional composition of individual active constituents (particularly QUE) in the ABR material or extract used was not performed. Methodologically, our work specifically centered on QUE’s modulation of the PI3K/Akt/eNOS axis and related phenotypic outcomes in NP cells, leaving its precise molecular targets and other predicted pathways functionally unvalidated. The experimental model relied exclusively on IL-1β stimulation, which simplifies the complex inflammatory, mechanical, and oxidative microenvironment characteristic of IDD. Although the RNA-seq analysis identified DEGs, it lacked subsequent pathway enrichment to interpret their functional networks. Although statistically significant results were obtained, the sample sizes remain modest, consistent with the preliminary nature of this work. Finally, essential translational parameters, notably the pharmacokinetic behavior and chronic toxicological profile of QUE, were not evaluated in this study.

Future studies should address these limitations through several complementary directions. First, the therapeutic effects of QUE require validation in AF and CEP cellular models to comprehensively evaluate its potential for the entire IVD unit. Concurrently, more physiologically relevant models incorporating combined cytokine exposure, mechanical loading, and oxidative stress would better recapitulate the multifactorial pathology of IDD. Second, a systematic investigation into the complete pharmacodynamic material basis of ABR is warranted. This should include quantitative profiling of QUE and other major active constituents using techniques such as HPLC or LC−MS/MS, followed by an assessment of their potential synergistic interactions, to clarify ABR’s holistic mechanism and compatibility principles. Third, broader functional screening should be conducted to validate additional pathways and the other hub targets, thereby constructing a more comprehensive network of QUE’s action in IDD. Direct assessment of PI3K/Akt/eNOS pathway activity in animal models would further substantiate QUE’s *in vivo* mechanism. Finally, expanding cohort sizes will enhance statistical reliability, while systematic pharmacokinetic and safety evaluations of QUE are essential for translational development. Collectively, these integrated approaches are expected to promote a systematic understanding of the TCM pharmacology in the treatment of IDD.

## 5 Conclusion

**Figure 10:**
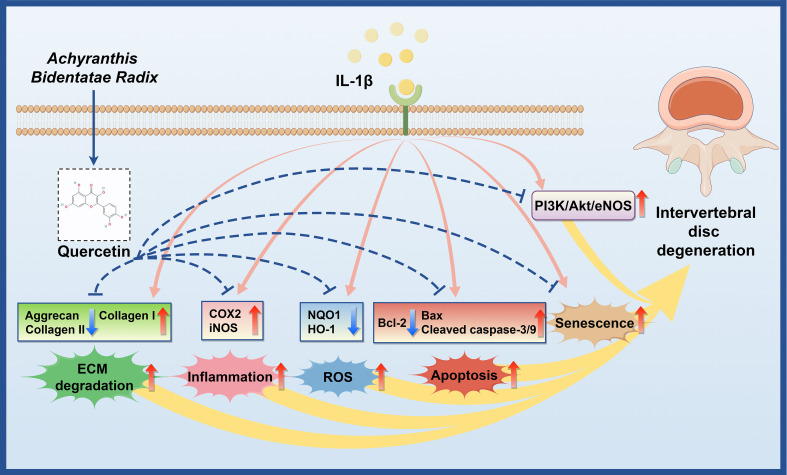
Schematic diagram of the potential therapeutic mechanism of ABR involved in IDD (by Figdraw 2.0).

Using an integrated network pharmacology and RNA-seq approach, we demonstrate that QUE, a key bioactive compound in ABR, effectively mitigates IL-1β-induced injury by regulating NP cell viability, oxidative stress, ECM degradation, inflammatory responses, apoptosis, and cellular senescence. *In vivo*, QUE significantly ameliorated IDD progression. Notably, this study is the first to establish that QUE exerts its protective effects in IDD through modulation of the PI3K/Akt/eNOS pathway (**Figure 10**). These findings elucidate a novel therapeutic mechanism of QUE, thereby paving the way for advancing ABR’s therapeutic potential in IDD treatment.

## Data Availability

The datasets presented in this study can be found in online repositories. The names of the repository/repositories and accession number(s) can be found below: HRA014617 (GSA; https://ngdc.cncb.ac.cn/gsa-human/browse/HRA014617) (93, 94).

## Glossary

IDD: Intervertebral disc degeneration
ABR: Achyranthis Bidentatae Radix
RNA-seq: RNA sequencing
MD: molecular dynamics
QUE: quercetin
NP: nucleus pulposus
IL-1β: interleukin-1β
ECM: extracellular matrix
IVD: intervertebral disc
AF: annulus fibrosus
CEP: cartilaginous endplates
TCM: Traditional Chinese Medicine
LBP: low back pain
DEGs: differentially expressed genes
OB: oral bioavailability
DL: drug-likeness
GO: Gene Ontology
KEGG: Kyoto Encyclopedia of Genes and Genomes
PPI: protein-protein interaction
FBS: fetal bovine serum
PS: penicillin/streptomycin
SPF: specific pathogen-free
MRI: magnetic resonance imaging
HE: hematoxylin and eosin
SO: Safranin O-fast green
SD: standard deviation
ANOVA: analysis of variance
BP: biological process
CC: cellular component
MF: molecular function
RMSD: root mean square deviation
RMSF: root mean square fluctuation
Rg: radius of gyration
NO: nitric oxide
NOS: nitric oxide synthase
