# Supplementary figures and images for: Exploring the mechanisms underlying quercetin, a key component of Achyranthis Bidentatae Radix, against intervertebral disc degeneration
Source: Front Immunol. 2026 Mar 10;17:1744969. doi: 10.3389/fimmu.2026.1744969 (PMC13008645; doi:10.3389/fimmu.2026.1744969)

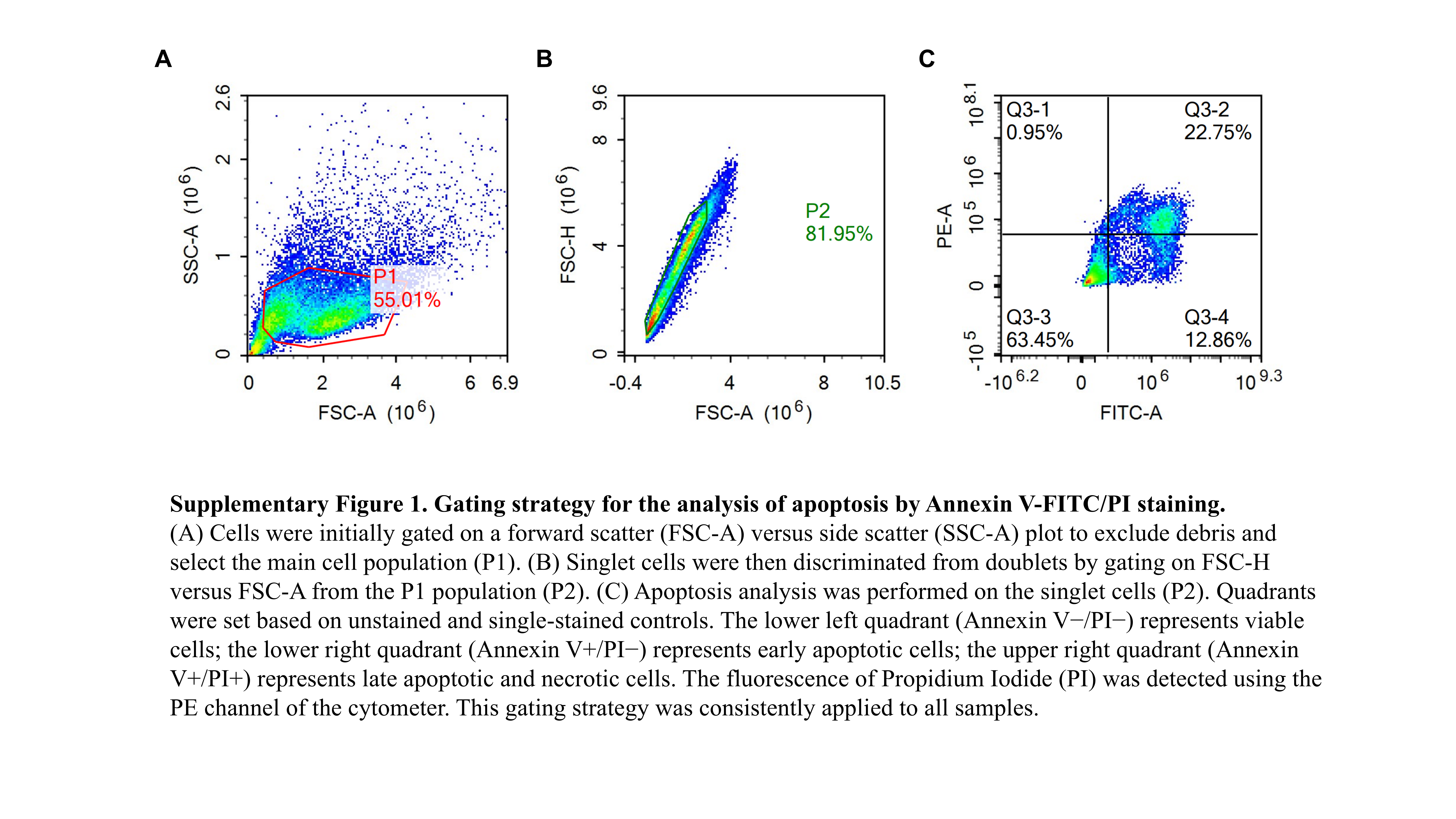

Supplement: Supplementary file 1 [file Image1.tif]

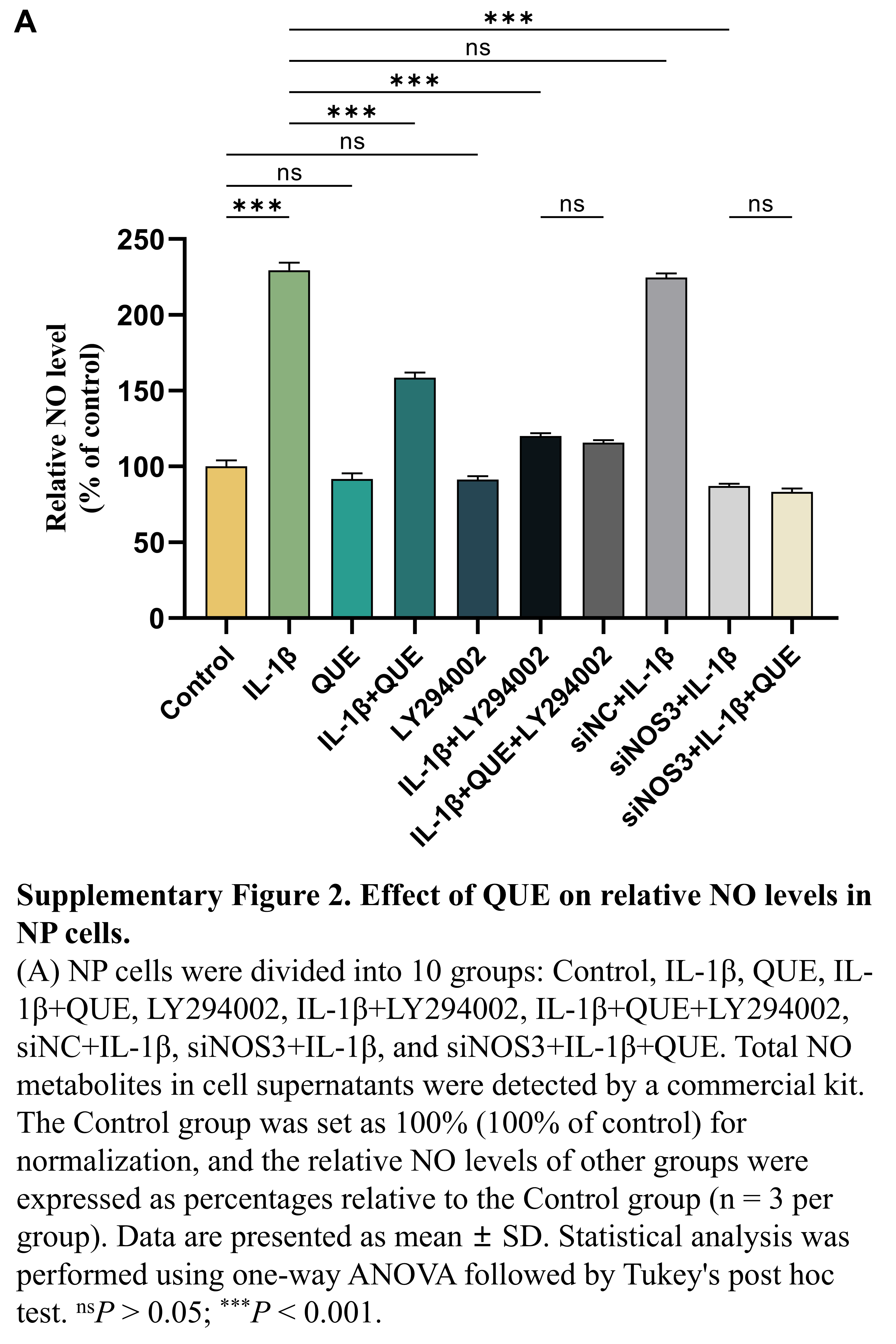

Supplement: Supplementary file 2 [file Image2.tif]
