# Supplementary material for: Exploring the mechanisms underlying quercetin, a key component of Achyranthis Bidentatae Radix, against intervertebral disc degeneration
Source: Front Immunol. 2026 Mar 10;17:1744969. doi: 10.3389/fimmu.2026.1744969 (PMC13008645; doi:10.3389/fimmu.2026.1744969)
Supplement: Supplementary file 3 [file Table1.doc]

**Supplementary Table S1. Clinical features of the patients.**

| Case | Age | Gender | Pfirrmann grade | Affected IVD |
| --- | --- | --- | --- | --- |
| 1 | 24 | Male | I | L4/5 |
| 2 | 28 | Female | I | L3/4 |
| 3 | 36 | Male | II | L4/5 |
| 4 | 41 | Male | IV | L4/5 |
| 5 | 44 | Female | IV | L5/S1 |
| 6 | 46 | Female | V | L4/5 |
