# Supplementary material for: Exploring the mechanisms underlying quercetin, a key component of Achyranthis Bidentatae Radix, against intervertebral disc degeneration
Source: Front Immunol. 2026 Mar 10;17:1744969. doi: 10.3389/fimmu.2026.1744969 (PMC13008645; doi:10.3389/fimmu.2026.1744969)
Supplement: Supplementary file 5 [file Table3.doc]

**Supplementary Table S3. Primers used in RT-qPCR analysis.**

| **Primers** | **Species** | **Forward (5’‒3’)** | **Reverse (5’‒3’)** |
| --- | --- | --- | --- |
| Nqo1 | Rat | TGAGAAGAGCCCTGATTGTATTG | CACCTCCCATCCTTTCTTCTTC |
| Hmox1 | Rat | CTGCTGACAGAGGAACACAA | CTGCAGAGGTAGTATCTTGAACC |
| Acan | Rat | GGTTCGAGTGAACAGCATCTAC | GGAGCGAAGGTTCTGGATTT |
| NOS3 | Rat | AAGCTGAGAGCCTGCAATTA | CTCTGTAGGTTCTCCACAGAAAG |
| GAPDH | Rat | TCTCTGCTCCTCCCTGTTCTA | GGTAACCAGGCGTCCGATAC |
