# Supplementary material for: Exploring the mechanisms underlying quercetin, a key component of Achyranthis Bidentatae Radix, against intervertebral disc degeneration
Source: Front Immunol. 2026 Mar 10;17:1744969. doi: 10.3389/fimmu.2026.1744969 (PMC13008645; doi:10.3389/fimmu.2026.1744969)
Supplement: Supplementary file 6 [file Table4.doc]

**Supplementary Table S4. Antibodies against the following proteins used in western bolt.**

| Antibody | Dilution | Catalog  Number | Company |
| --- | --- | --- | --- |
| NQO1 | 1:1000 | A23486 | ABclonal |
| HO-1 | 1:1000 | A1346 | ABclonal |
| Collagen I | 1:2000 | A22090 | ABclonal |
| Aggrecan | 1:1000 | A11691 | ABclonal |
| eNOS | 1:1000 | A20985 | ABclonal |
| Phospho-eNOS | 1:500 | AP0516 | ABclonal |
| PI3K | 1:1500 | A4992 | ABclonal |
| Phospho-PI3K | 1:1000 | AP0427 | ABclonal |
| GAPDH | 1:50000 | A19056 | ABclonal |
| Collagen II | 1:1000 | ab307674 | Abcam |
| iNOS | 1:1000 | ab178945 | Abcam |
| COX2 | 1:1000 | ab179800 | Abcam |
| Bax | 1:1000 | ab32503 | Abcam |
| Akt | 1:1000 | 2938 | CST |
| Cleaved caspase-3 | 1:1000 | 9661 | CST |
| Cleaved caspase-9 | 1:1000 | 9507 | CST |
| Phospho-Akt | 1:1000 | 4051 | CST |
| Bcl-2 | 1:1000 | 26593-1-AP | Proteintech |
| Goat anti-Mouse IgG Antibody Peroxidase ConjugaTed ,H+L | 1:5000 | AP124P | Millipore |
| Goat anti-Rabbit IgG (H+L) Secondary Antibody, HRP | 1:10000 | 31460 | Thermo |
