# Supplementary material for: Exploring the mechanisms underlying quercetin, a key component of Achyranthis Bidentatae Radix, against intervertebral disc degeneration
Source: Front Immunol. 2026 Mar 10;17:1744969. doi: 10.3389/fimmu.2026.1744969 (PMC13008645; doi:10.3389/fimmu.2026.1744969)
Supplement: Supplementary file 8 [file Table6.doc]

**Supplementary Table S6. Main active ingredients in ABR.**

| Mol ID | Molecule Name | OB (%) | DL |
| --- | --- | --- | --- |
| MOL001006 | poriferasta-7,22E-dien-3beta-ol | 42.98 | 0.76 |
| MOL012461 | 28-norolean-17-en-3-ol | 35.93 | 0.78 |
| MOL012485 | achyranthoside c_qt | 66.62 | 0.18 |
| MOL012505 | bidentatoside,ii_qt | 31.76 | 0.59 |
| MOL012537 | Spinoside A | 41.75 | 0.4 |
| MOL012542 | β-ecdysterone | 44.23 | 0.82 |
| MOL001454 | berberine | 36.86 | 0.78 |
| MOL001458 | coptisine | 30.67 | 0.86 |
| MOL000173 | wogonin | 30.68 | 0.23 |
| MOL002643 | delta 7-stigmastenol | 37.42 | 0.75 |
| MOL002714 | baicalein | 33.52 | 0.21 |
| MOL002776 | Baicalin | 40.12 | 0.75 |
| MOL002897 | epiberberine | 43.09 | 0.78 |
| MOL000358 | beta-sitosterol | 36.91 | 0.75 |
| MOL003847 | Inophyllum E | 38.81 | 0.85 |
| MOL000422 | kaempferol | 41.88 | 0.24 |
| MOL004355 | Spinasterol | 42.98 | 0.76 |
| MOL000449 | Stigmasterol | 43.83 | 0.76 |
| MOL000785 | palmatine | 64.6 | 0.65 |
| MOL000085 | beta-daucosterol_qt | 36.91 | 0.75 |
| MOL000098 | quercetin | 46.43 | 0.28 |
